# A thermosurvey dataset: Older adults’ experiences and adaptation to urban heat and climate change

**DOI:** 10.1038/s41597-024-03509-4

**Published:** 2024-06-22

**Authors:** Barbara Jancewicz, Małgorzata Wrotek

**Affiliations:** University of Warsaw, Warsaw, Poland

**Keywords:** Environmental health, Sociology, Economics

## Abstract

We introduce the thermosurvey dataset, a comprehensive collection focusing on the thermal comfort, heat-related experiences, health, socioeconomic status, and perceptions of older adults (aged 65 and over) in Warsaw and Madrid. The two cities differ greatly in their heat experiences, but due to climate change, both face increasing temperatures. The study aimed to understand how heat affects cities’ older adult population and how we can better adapt to rising temperatures. We call the study a thermosurvey because it connects traditional survey data with temperature and humidity measurements done before, after and during the interview, offering a holistic view of the participants’ thermal environments. The dataset can be used to better understand thermal comfort, the interplay of health and heat experiences, and the relationship between experiences and climate change views. We hope our data will enable scholars to analyse the impact of climate change on older adults and to develop strategies to help them adapt to a warming climate.

## Background & Summary

The growing impact of climate change, while universal, manifests unevenly across different regions and populations^1,2^. Nevertheless, for the broader public climate change remains an abstract phenomenon weakly related to their everyday life. Furthermore, while views on climate change are a common topic of social science studies^3,4^ and policies related to climate change are widely discussed in political science literature, research on the impact of climate change is still dominated by the natural sciences^5^. Our study, and more generally the project “Embodying Climate Change: Transdisciplinary Research on Urban Overheating”, aims to change that by studying the relationship between climate change and people’s everyday lives. In this study, we focused on heat, as one of the most known results of climate change and simultaneously one that is difficult to touch, smell or see. Therefore, the survey presented here investigated people’s heat related experiences, opinions, health preconditions, as well as thermal comfort set against measured temperatures.

The project focused on urban areas, particularly vulnerable to climate risks due to dense populations and infrastructure^6^. In particular, we conducted fieldwork in two European cities because Europe is becoming a heatwave hotspot^7^. We selected Madrid and Warsaw for our fieldwork, as they represent contrasting climatic histories while they both face escalating heat challenges. Madrid with a long experience of high temperatures, that now increase further; and Warsaw with a history of cold winters and mild summers, which is now transitioning into an even warmer future.

Within each city, we further zoomed in on the most vulnerable group, not the young people –who are commonly studied since they are expected to bear the brunt of climate change – but the older adults (65 years old or older), who are at constant risk both regarding their health and wellbeing. Additionally, the population of older adults is growing and is projected to increase further with those who were previously young ageing and reaching old age^8,9^.

The presented survey is a unique large-scale study of the urban older adult population of two European cities Warsaw and Madrid. The study also contains another innovative aspect: it connects traditional survey data (questions regarding housing conditions, lifestyle and physical activity, health, independence with ADL and iADL scales, thermal preferences, thermal comfort measured in line with the PN-EN ISO 10551 norm (the 2019 update), heat-related experiences and strategies, climate change views, preferences regarding long-term care, socioeconomic status) with temperature and humidity measurements done before and during the interview. The overall result is a thermosurvey.

Our research aims to contribute to a deeper understanding of how heatwaves affect older adults in urban settings and to inform strategies for adapting to rising temperatures. This is in line with research underscoring the potential of physiological, behavioural, infrastructural, and technological adaptations in mitigating the adverse effects of heat^10^. By sharing our data, we hope to facilitate further research into effective adaptation mechanisms for urban populations amid escalating climate challenges.

## Methods

### The studied population

We studied the population of older adults – people aged 65 or older – who at the time of the study lived in the city bounds of Warsaw or Madrid. Within that population, we limited our sample to only those people who were healthy and cognitively active enough to give informed consent and answer the interviewers’ questions.

### Sampling

To provide a diversified representation of older adults the study used a quota sample. The quotas were based on three variables: the city (Madrid/Warsaw), respondent’s sex (male/female) and age (65–69,70–74,75–79,80–84,85+). These variables defined 20 quota groups, and for each group, 100 interviews were planned. Additionally to the set quotas, the interviewers also tried to diversify city districts in which the respondents lived to provide better geographical coverage. Furthermore, only one person from a household could take part in the study.

### Respondents’ recruitment

Respondents were invited to participate in the study both by the interviewers and by the study coordinators in each city. Throughout the summer the study coordinators posted ads in traditional media and social media, made telephone calls and sent or delivered letters to potential participants. Interviewers were limited in their ability to recruit respondents because they could not ask people in public spaces (streets, parks, shopping centres etc.) nor in typical gathering places (senior clubs, churches, hospitals etc.). Such recruitment strategies could skew the sample towards people in one location and with similar lifestyle. It could also encourage conducting the interview on the spot, never visiting the respondent’s apartment, and remaining in public, which could impact answers and respondents’ comfort. Thus, we limited this type of recruitment to ensure respondents’ privacy and gather a more diversified sample. Interviewers could rely on door-to-door recruitment, and they could support the coordinators’ efforts via calling or delivering letters.

Initially, all older adults were invited to participate. When the number of interviews arranged in a quota group exceeded 100 then new interviews for that group were no longer arranged by the study coordinators. Interviewers, who could recruit independently and arrange their interviews, were regularly informed about current quotas. Even when a quota was filled, the already arranged interviews were still conducted leading to some quotas exceed 100. Final interview back-checks and consistency analysis led to the removal of some interviews causing some quotas to fall slightly below 100. As Table 1 shows, overall, we conducted between 91 and 138 interviews in each quota group providing sufficient representation and enabling group comparisons.

**Table 1:** Survey respondents by their characteristics used to create quota groups: city, age, and sex.

City, Sex/Age Group	Warsaw		Madrid	
	Males	Females	Males	Females
65–69	110	120	100	102
70–74	107	108	101	103
75–79	105	107	138	97
80–84	101	101	114	101
85+	100	91	102	103
Sum	1050	1061		

### Ethics approval

The Centre of Migration Research Ethics Committee first advised postponing the study from summer 2021 to 2022 in order to better safeguard interviewers and study participants from a possible COVID-19 infection. The study obtained the Ethics Committee approval (reference number CMR/EC/10/2021) for running the survey throughout the summer of 2022.

### Data collection

The survey took place in 2022 during an exceptionally hot summer. During that summer Madrid experienced 60 days and Warsaw 26 days with temperatures so high, that the municipalities issued heatwave alerts^11^. The first interview took place on 2 July 2022 and the last on 15 September 2022. Overall, 2111 interviews were conducted (1061 in Madrid and 1050 in Warsaw).

The responses were collected by conducting Computer Assisted Personal Interviews (CAPI). The interviewers visited respondents and used a tablet and an online form to fill out the survey, and when necessary, a handheld compass to judge the directions of the windows in respondents’ home or apartment, and the UNIT-T UT333 Mini Temperature Humidity Meter to measure temperature and humidity at the beginning, middle and the end of the interview.

### Research tool

The survey consisted of several distinct parts. It started with three introductory sections. First “Identification data” with basic localization and demographic information. Then, there was space for “Interviewer’s Observations” regarding respondents’ building as well as the first measurement of temperature and humidity. The last of these sections called “Introduction” contained a script to talk to the study participant, inform him/her about the study and confirm their willingness to take part.

This introductory part was followed by three sections establishing the respondents’ background situation. It started with questions regarding “I. Housing Conditions” where respondents talked about their home, how long they lived in the city or in the apartment/house, how big it is, where do the windows face and what kind of amenities it has. Some questions regarding building conditions correspond to or were modified based on SHARE survey^12^. Then the survey transitioned to inquiring about “II. Lifestyle and Physical Activity” with questions about sports, smoking and selected four questions from health-related quality of life questionnaire SF-36 (stair climbing, bending down, walking 100 and 1000 m)^13^. Then participants self-reported their “III. Current Health” including their self-rated health (the question originally proposed by Ware and Sherbourne^14^, then modified in the PolSenior study^15,16^ to include a 0–10 scale, and then by us by adding colour to the scale), COVID-19 history, and a list of questions regarding common ailments that could impact their heat experience e.g., cardiovascular problems, hyperthyroidism, depression. The section closes with queries on weight, height, and fluid intake. The fourth main section “IV. Independence” included ADL^17^ (abilities such as bathing, dressing, transferring, feeding, incontinence, toileting) and iADL^18^ (abilities such as using a telephone, getting to places beyond walking distance, shopping, preparing meals, housework, washing, taking medications, money managing) scales with some modifications inspired by the PolSenior study^15,16^ and closed off with a question about regular internet usage.

The background part was followed by the focus of the study: “V. Experiencing Heat” subdivided into 4 sections. The first section called “A. Feeling the Temperature Now” helped respondents to reflect on their temperature preferences and current bodily feelings using colour scales. This section contains four questions that follow the PN-EN ISO 10551 norm to measure thermal comfort. After evaluating the temperature experiences participants were asked to evaluate whether inside (or outside, depending on where the interview took place) was cooler or warmer. Finally, the interviewer noted the current readings of the temperature and humidity meter.

The second section related to experiencing heat dealt with past experiences of heat (“B. Feeling the Latest Heat”), it started by asking participants to recall the last heatwave and evaluate how they felt inside and outside. Then they were asked a set of 25 questions regarding different feelings, symptoms, or experiences such as thirst, sweating, fatigue, chest pain, headache, and irritation. First, we asked whether they had or experienced them. Second, if they answered yes, then we asked whether this feeling/symptom/experience happens more often or is stronger during heat.

The third section related to experiencing heat focused on “C. Strategies”: ways of dealing with the temperatures. The section started with questions on where they spend more or less time during heat and then followed with a list of possible strategies (drawn from initial results of the qualitative parts of the “Embodying Climate Change. Transdisciplinary Research on Urban Overheating” project) that they use to keep themselves cool during heat.

The last section related to experiencing heat asked about “D. Perceptions of Climate Change”. First participants indicated where they get weather information from, then they evaluated their level of worry with occurrences of heat, and whether they think that the summers are getting hotter. Then we asked about belief in recent climate change, and if the bouts of heat are related to it. Finally, we asked the European Social Survey 8 wave’s^19^ question on causes of climate change (natural processes vs. human activity), level of worry about climate change and beliefs on whether it impacts their daily life and health. The section ended with questions about recent changes in their environment regarding green and blue spaces, as well as the amount of climate change information.

After closing the sections concerned with experiencing heat the survey transitioned to establishing what types of “VI. Formal and Informal Care” respondents could rely on. Thus, the section asked about household and family composition, frequency of contacts, and their capabilities and preferences regarding long-term care. The interview finished by asking participants about their “VII. Basic Data” such as place of birth, education, work history, apartment/house ownership, income, and political leaning. Finally, the interviewer measured the temperature and humidity for the last time and thanked for the interview. After saying goodbye to the respondent, it was the interviewer’s turn to answer several questions about the interview in the section “VIII. Implementation and Evaluation of the Interview Process”.

### Data anonymization

Our research followed the data minimisation principle, trying to gather only the information we considered crucial to our study aims. Still, the original data gathered included variables that potentially enabled respondents’ reidentification. Therefore, the dataset was thoroughly anonymised before sharing. First, we conducted a risk analysis by considering how variables in our dataset could be combined with other data sources to de-anonymise study participants. We considered both publicly available satellite images, land and mortgage registers and data published by the statistical offices (e.g. census data); as well as databases with limited access, such as records held by healthcare providers and registry offices (births, marriages etc.). We tried to cross reference the contents of these registers with our respondent’s answers taking into consideration their precision, truthfulness, timeliness, and uniqueness among other older adults in both cities.

Following this risk analysis, we first focused on anonymising the building in which the respondent lived at the time of the interview. We removed information about interview location (GPS and postal code) leaving only the city district. We grouped the type of building in which respondents live and removed the additional explanation in the “other” category, to anonymise unique building types. We grouped the information about apartments’ floor level, and the year in which the buildings were built according to study participants, so that neither the height nor the age of the building would enable identification. Furthermore, we grouped the size of the apartments/houses, so that the building cannot be identified thanks to the size characteristic for a certain building design. Additionaly, we removed or grouped information about visible (inbuilt AC) or rare (pool) amenities. Finally, we also removed the information about the directions that the windows face, leaving only a variable that reports whether at least one window faced south, south-west or south-east, to account for sun exposure. Thus, even after linking full land registers with in-depth satellite imaging analysis, one would not be able to pinpoint the respondent’s building.

The second part of anonymisation focused on the individual. The biggest change is aggregating the age of study participants into five-year groups. We also removed information when a person moved to a certain apartment, as well as the descriptions of non-family cohabitants, in order to remove a possible link to the residence registers. Similarly, we removed the description of people that were present during the interview. We capped the number of sons, daughters, grandsons, and granddaughters at four, to protect the anonymity of larger families. Furthermore, if a respondent was born in a different country than the one they lived in, we removed the country of birth, so that it would not identify them. We broke possible links to the tax office and social insurance institutions’ data, be removing personal income, grouping the information on real estate ownership and on years of employment. We did retain respondents’ evaluation of their household financial situation. The remaining answers, have the anonymising benefit of being self-reported (thus sometimes different than official registers), of being recorded almost two years before the data was shared, and of the study participants belonging to a large population of older adults that reach almost two million in Warsaw and over three million in Madrid.

Throughout our research we followed the credo “as open as possible and as closed as necessary”. Thus, we tried to retain as much data as possible, to enable better understanding of the relationships between heat, health, and older adults, while protecting our respondents’ anonymity. Therefore, after initial risk assessment and anonymisation we asked the CMR Ethics Committee to evaluate our efforts – we received recommendations, which we applied, and then the Committee deemed the data anonymous. Finally, we received reviews from the Scientific Data’s Reviewers and in reaction we decided to anonymise the data even further. Therefore, we consider the data anonymised.

## Data Records

The dataset and all the metadata in English have been deposited in Social Data Repository^20^ (rds.icm.edu.pl): 10.18150/JZZ7NR. All the files are also available in Polish in a twin entry here^21^: 10.18150/XRWJKS.

The dataset and frequency tables are shared under the Creative Commons Attribution 4.0 (CC BY) licence, while the accompanying questionnaire and other metadata are under the Creative Commons 4.0 (CC) licence. The first file is a pdf containing the dataset’s detailed documentation: 1. Description of the dataset, 2. Codebook, 3. Questionnaire in English (translation), 4. Questionnaire in Polish (used in Warsaw), 5. Questionnaire in Spanish (used in Madrid), 6. Questionnaire Instruction for Interviewers that explained how to ask or interpret the trickier of the survey’s questions.

The dataset itself is shared in two formats:

- .sav (IBM SPSS Statistics) where it is accompanied by variable and value labels;
- .csv (a semicolon-separated file)

After the dataset, the entry also includes the frequency tables requested by the Repository. The last file is a Microsoft Excel file containing a codebook: variable and value labels.

### Weights

The dataset contains post-stratification weights that use city (Warsaw, Madrid), age (65–69, 70–74, 75–79, 80–84, 85+) and sex (male, female) to adjust the sample’s structure to 2021 population censuses results. The highest weight is 1.64 for females aged 65–69 in Warsaw, and the lowest is 0.45 for men aged 80–84 in Warsaw (Table 2). After applying weights, the age and sex structure of the sample follows closely the 2021 structure of the older adults’ population in each city.

**Table 2:** Survey post-stratification weights based on respondents’ city, age and sex.

City, Sex/Age Group	Warsaw		Madrid	
	Males	Females	Males	Females
65–69	1.27	1.64	1.08	1.40
70–74	0.98	1.48	0.95	1.31
75–79	0.54	0.93	0.59	1.26
80–84	0.45	0.90	0.54	1.02
85+	0.47	1.19	0.65	1.41

## Technical Validation

### Respondents’ characteristics

**Fig. 1:**
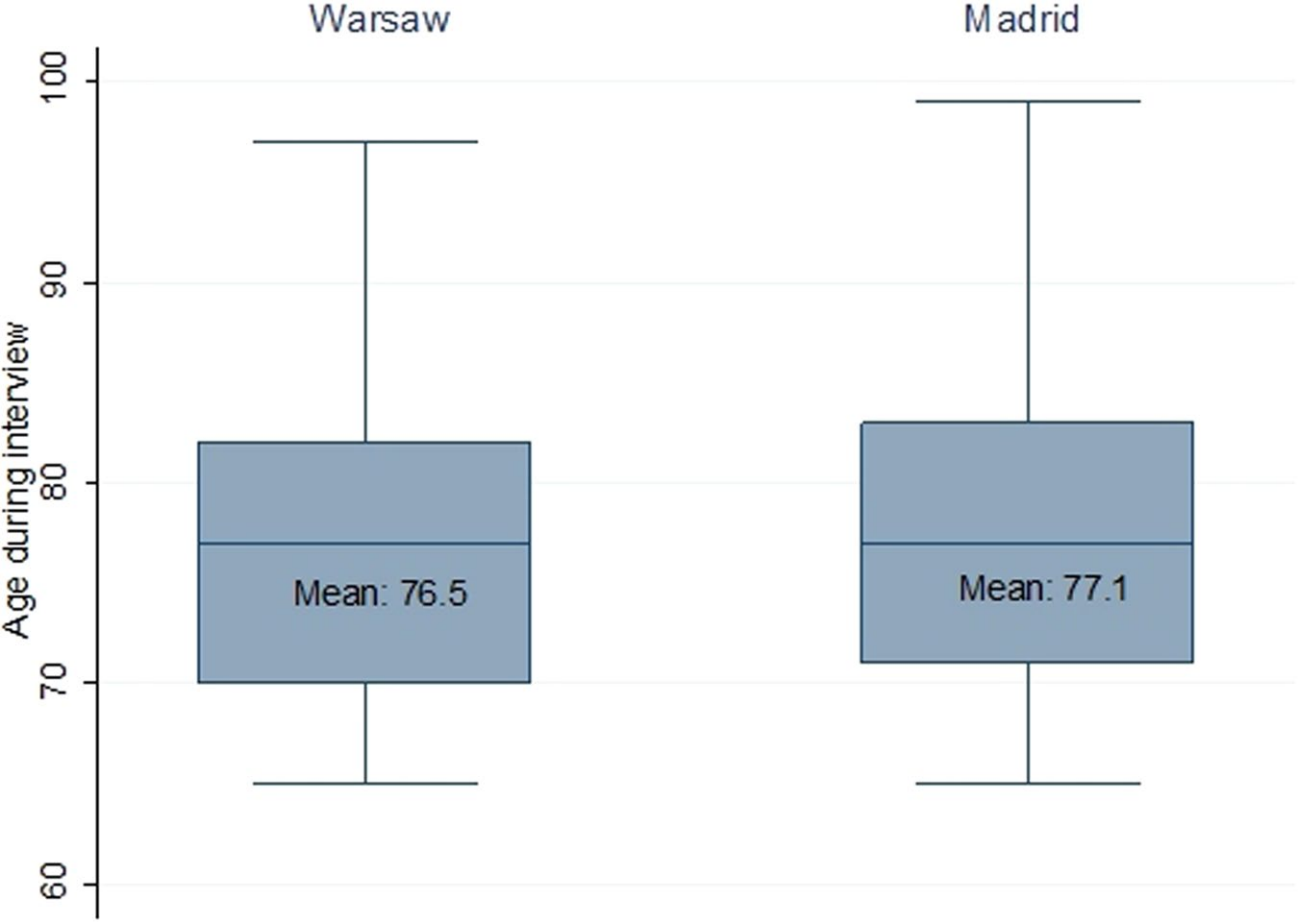
Respondents’ age distribution by the city of interview (sample N = 2111, unweighted data).

The equal distribution of the sample in terms of age and gender ensured that the mean age of respondents for both cities was similar: 76.5 for Warsaw and 77.1 for Madrid (Fig. 1). The quota group recruitment made it possible to obtain a comparable proportion of responses from adults 85 and older, who are usually underrepresented in surveys.

**Fig. 2:**
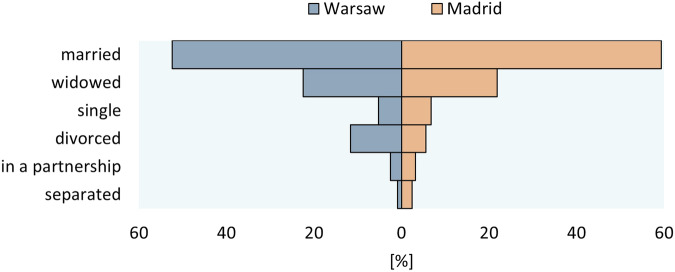
Respondents by their marital status and city of interview (sample N = 1050 Warsaw and 1061 Madrid, unweighted data). Note: Refusal to answer/hard to say: 52 (4.94%) in Warsaw and 8 (0.75%) for Madrid.

The distribution of marital status among respondents was similar in the two cities (Fig. 2) with the majority of participants in both Warsaw and Madrid being married (52.4% and 59.4% respectively). The second most numerous group, one-fifth of all respondents, consists of widows and widowers (22.5% in Warsaw and 21.9% in Madrid).

**Fig. 3:**
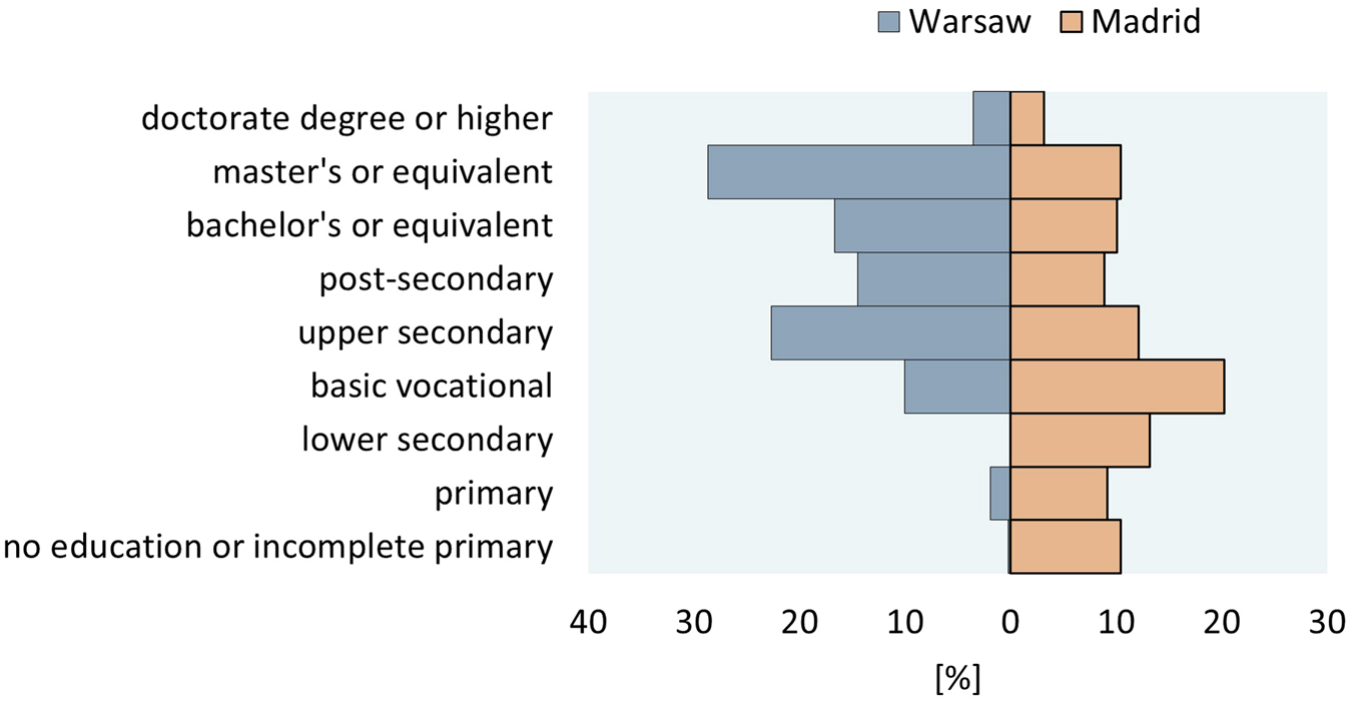
Respondents by their highest level of education and by city (sample N = 1050 Warsaw and 1061 Madrid, unweighted data). Note: Refusal to answer/hard to say: 20 (1.9%) in Warsaw and 23 (2.18%) for Madrid.

Warsaw and Madrid’s samples differed in their education levels’ distribution (Fig. 3). In Warsaw participants with secondary and tertiary education dominated, while in Madrid participants were more evenly distributed between education levels. The difference is most visible among respondents with primary or lower education, which was reported by almost 20% of Madrid’s respondents but only by 2% of Warsaw’s respondents. Such differences can result from respondents’ selection or from differences in the underlying populations. Unfortunately, at the time of the survey and data release, the latest data on the educational distribution of the population of both cities from Censuses was not available. Hence, to account for this characteristic of the dataset, it is recommended to include education level in the analysis and interpretation of results from the study.

### Interviews’ characteristics

**Fig. 4:**
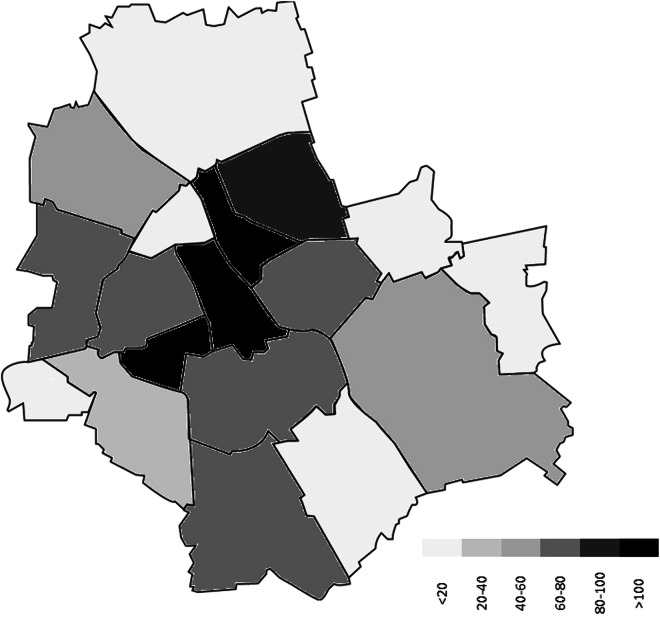
Map of Warsaw and the number of interviews by city district of the interview location (district’s shape taken from https://zdm.waw.pl/miejski-system-informacji/obszary-msi/).

**Fig. 5:**
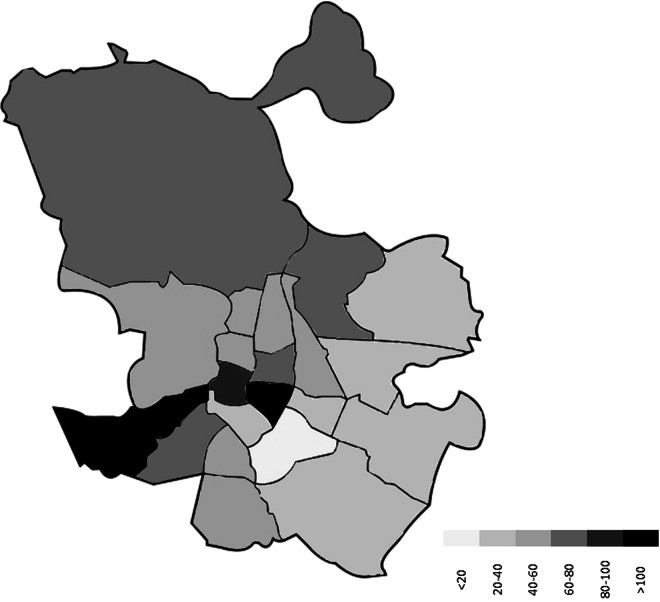
Map of Madrid and the number of interviews by city district of the interview location (district’s shape taken from https://images.app.goo.gl/ie9tLtN5627Ezfo19).

The maps (Figs. 4, 5) show the distribution of interviews between each city’s districts. Both the number of interviews and the size of the older adult population vary between the city districts, they are however not in sync. Thus, the dataset does not provide a representative coverage of geographical variations within the cities. However, it is worth noting, that more interviews were carried out closer to city centres, where the share of older adults is usually higher and where the urban heat island effect is usually stronger than in other areas. Thus, one could consider dividing the data into two or three city district groups and with proper weighting compare results for areas with different characteristics.

**Fig. 6:**
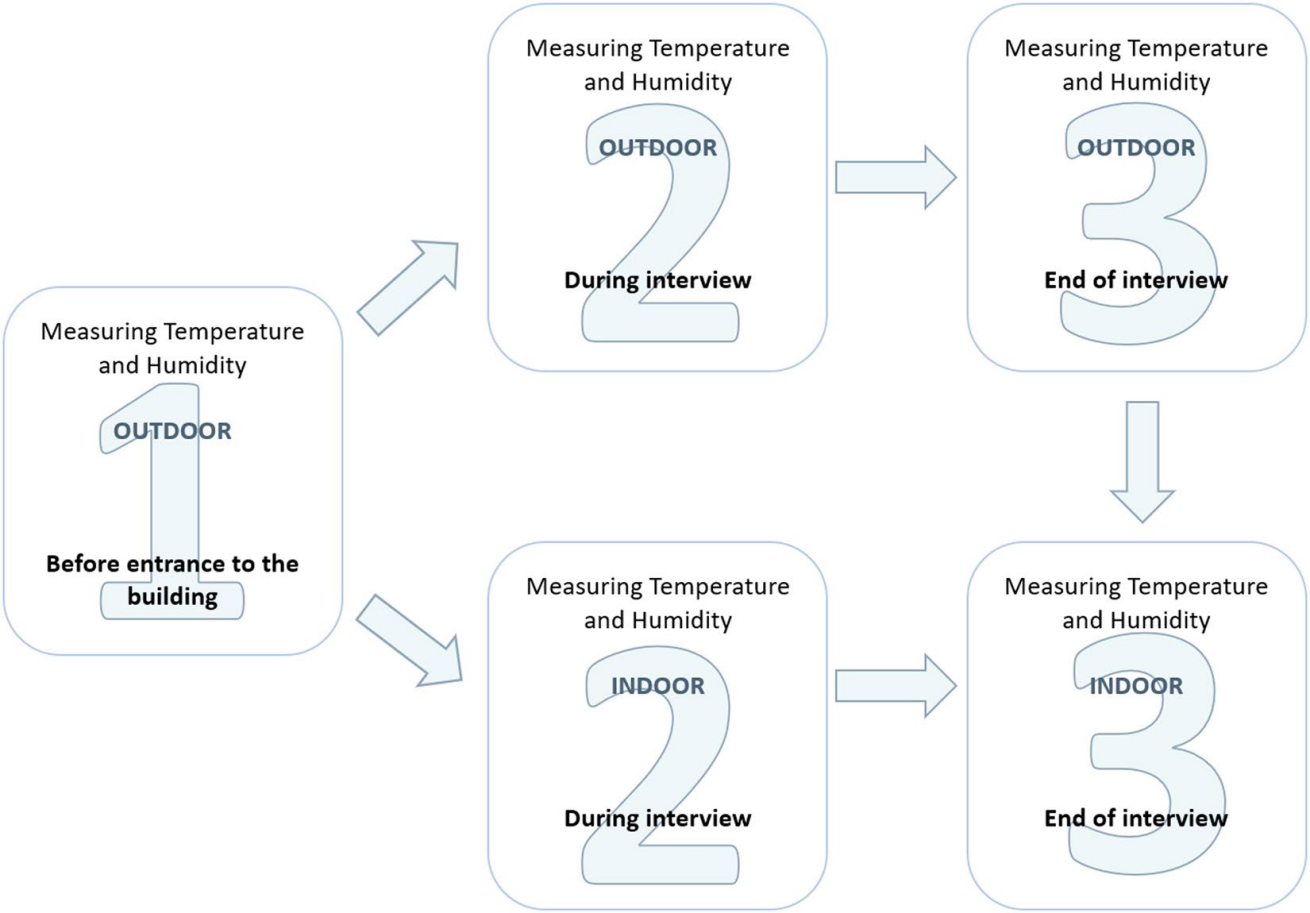
The temperature and humidity measurement procedure used in the study.

Interviewers took temperature and humidity measurements three or four times during each interview (Fig. 6). The first measurement took place before entering the building, the second during the interview, and the third at the end of the interview. Following the ethics committee recommendation in the study, we enabled participants to decide whether they wanted to take the interview inside or outside. As a result, 37.6% of interviews in Warsaw and 27.9% of interviews in Madrid took place outside the respondent’s home. Thus, in such cases to collect temperature measurements also from inside the home, the last temperature measurement was taken twice: once outside and once inside the home. More precisely, when at the beginning of the interview respondents declared that they wanted to take the interview outside, they were asked to leave one thermo-hygrometer inside the home for the duration of the interview, and then at the end of the interview, they were asked to freeze or record the measurement and return the meter to the interviewer.

**Fig. 7:**
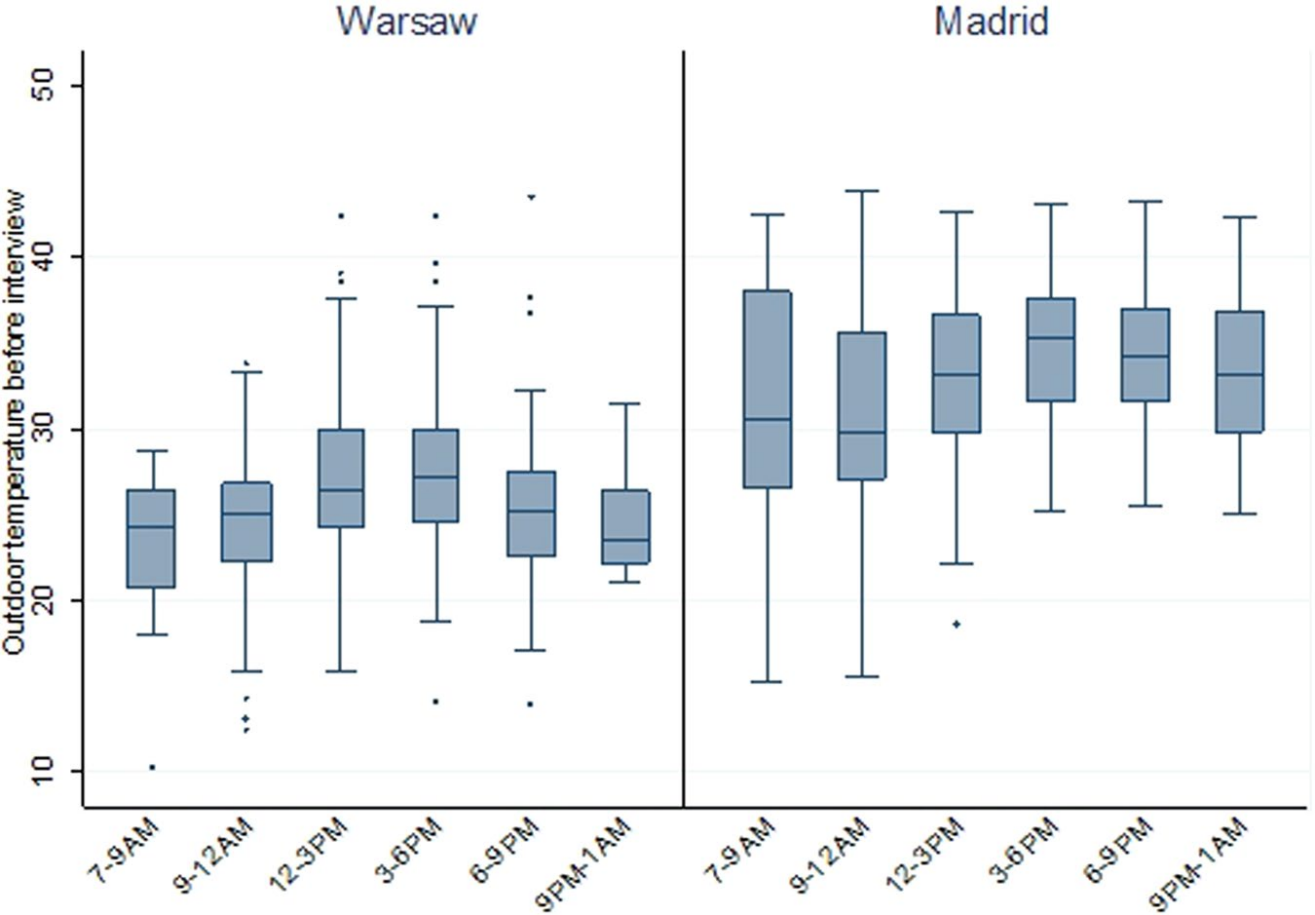
Distribution of outdoor temperature readings before starting the interview by time intervals (sample N = 1050 Warsaw and 1061 Madrid, unweighted data).

Temperatures fluctuate throughout the summer and throughout the day. Although the survey was not designed to focus interviews only during the hottest times of day, the majority of interviews were conducted at times with higher temperatures (Fig. 7). Table 3 showcases that most interviews took place between noon (12 PM) and 9 PM (82.57% in Warsaw and 64.85% in Madrid). This distribution enables better inference of heat-related experiences by older people in both cities.

**Table 3:** Number of interviews by time intervals and mean value of outdoor temperature readings before interview by cities (sample N = 1050 Warsaw and 1061 Madrid, unweighted data).

Time interval	Warsaw		Madrid	
	Per cent	Mean temp.	Per cent	Mean temp.
7:00 AM-9:00 AM	1.4	23.1	3.7	31.5
9:00 AM-12:00 PM	14.8	24.5	17.9	30.7
12:00 PM-3:00 PM	30.6	26.9	21.8	33.1
3:00 PM-6:00 PM	32.6	27.1	19.2	34.6
6:00 PM-9:00 PM	19.4	25.1	23.9	34.1
9:00 PM-1:00 AM	1.2	24.6	13.6	33.3
Total	100	26.2	100	33.1

## Usage Notes

This dataset provides a unique focus on the ways people perceive and experience heat. It also fills the gap of limited research data on older adults, their views, socioeconomic situation, health, and experiences. Furthermore, aside from people’s experiences of heat and selected apartment characteristics, it also contains temperature and humidity measurements. The measurements make the study a perfect dataset to analyse factors that determine the apartments’ temperature or what impacts older adults’ thermal comfort.

**Fig. 8:**
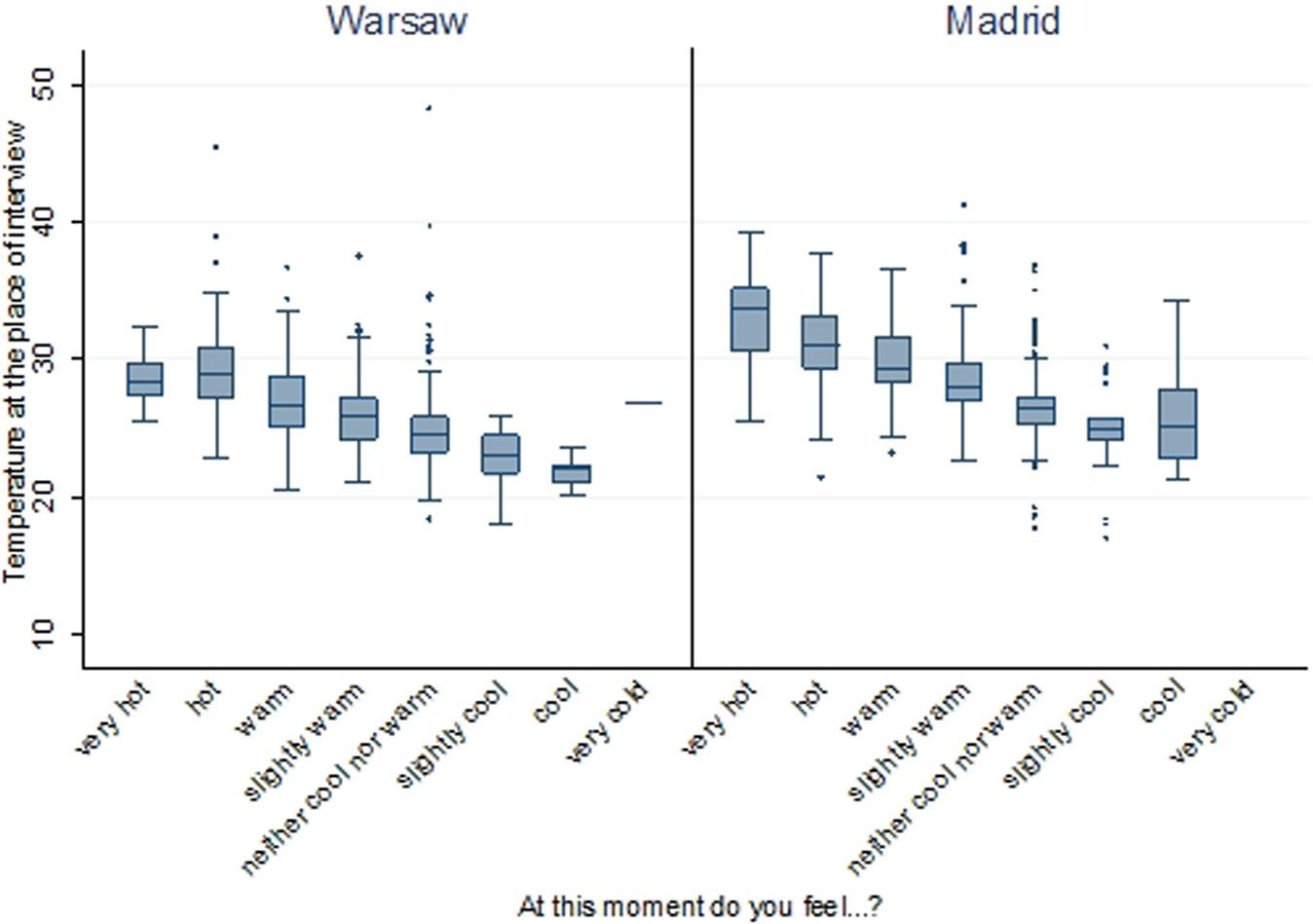
Temperature readings at the place and time of interview vs. temperature experience based on the PN-EN ISO 10551 norm (sample N = 1043 Warsaw and 1056 Madrid, unweighted data). Note: The category “very cold” includes only 1 observation in Warsaw.

For example, the questions in the survey, combined with the temperature measurements, allow us to assess older people’s individual exposure to and experiences of heat. Figure 8 shows, that while there is a strong relationship between the temperature and how respondents evaluated, we also see that older people experience the temperature differently in the two cities, which confirms residents’ acclimatization to their local climate^22^. Similar as well as more granular analyses can be conducted using our dataset.

Furthermore, extensive health sections of the questionnaire enable analyses of how health translates into heat experiences, adaptation measures and climate change views. Data on the frequency of contacts, household structure and respondents’ independence make it possible to gauge their vulnerability in a case of crisis, be it heat, or non-heat-related. These are the several uses that we considered most likely, but we are convinced that the research community will surprise us with creative usage of the dataset to better understand how heat impacts people.

## Data Availability

The study did not use any custom code to generate data.
